# Diagnosing arsenic-mediated biochemical responses in rice cultivars using Raman spectroscopy

**DOI:** 10.3389/fpls.2024.1371748

**Published:** 2024-03-25

**Authors:** Isaac D. Juárez, Tianyi Dou, Sudip Biswas, Endang M. Septiningsih, Dmitry Kurouski

**Affiliations:** ^1^ Department of Biochemistry and Biophysics, Texas A&M University, College Station, TX, United States; ^2^ Interdisciplinary Faculty of Toxicology, Texas A&M University, College Station, TX, United States; ^3^ Department of Soil and Crop Sciences, Texas A&M University, College Station, TX, United States

**Keywords:** *Oryza sativa*, phenylpropanoids, carotenoids, stress pathways, non-invasive analysis, analytical techniques, bioaccumulation

## Abstract

Rice (*Oryza sativa*) is the primary crop for nearly half of the world’s population. Groundwater in many rice-growing parts of the world often has elevated levels of arsenite and arsenate. At the same time, rice can accumulate up to 20 times more arsenic compared to other staple crops. This places an enormous amount of people at risk of chronic arsenic poisoning. In this study, we investigated whether Raman spectroscopy (RS) could be used to diagnose arsenic toxicity in rice based on biochemical changes that were induced by arsenic accumulation. We modeled arsenite and arsenate stresses in four different rice cultivars grown in hydroponics over a nine-day window. Our results demonstrate that Raman spectra acquired from rice leaves, coupled with partial least squares-discriminant analysis, enabled accurate detection and identification of arsenic stress with approximately 89% accuracy. We also performed high-performance liquid chromatography (HPLC)-analysis of rice leaves to identify the key molecular analytes sensed by RS in confirming arsenic poisoning. We found that RS primarily detected a decrease in the concentration of lutein and an increase in the concentration of vanillic and ferulic acids due to the accumulation of arsenite and arsenate in rice. This showed that these molecules are detectable indicators of biochemical response to arsenic accumulation. Finally, a cross-correlation of RS with HPLC and ICP-MS demonstrated RS’s potential for a label-free, non-invasive, and non-destructive quantification of arsenic accumulation in rice.

## Introduction

As the human population grows, food demand increases proportionally. In just under 30 years, the need for food is estimated to increase by 30% (Agriculture USDo Food Security). To meet the growing food demands, numerous strategies are used in farming, including genetically modified plants and timely diagnostics of biotic and abiotic stresses (Jiehua et al., 2019; Rusinque et al., 2021). Among these stressors, arsenic ions, including arsenite (As^+3^) and arsenate (As^+5^), are among the greatest threats to human health. Arsenate is more commonly found in groundwater compared to arsenite (Arsenic, metals, fibres, and dusts, 2012), although their ratio is dependent on water conditions, notably pH and the presence of oxygen. In some parts of Southeast Asia, both naturally occurring and anthropogenic arsenites and arsenates contaminate the groundwater at levels toxic to human health (Kim et al., 2011). This same groundwater is used for irrigation of rice paddies, where the anaerobic environment of the rice paddy soil readily renders arsenic ions available (Signes-Pastor et al., 2016). The combination of irrigating rice paddy soils with arsenic-polluted water and rice’s inherent plant physiology results in its accumulation of arsenic at levels 10-20 times higher than other grains (Nunes and Otero, 2017; Liao et al., 2018). In rice, arsenate mimics phosphate and is heavily taken up by phosphate transporters, while arsenite is primarily transported by nodulin 26-like intrinsic proteins (NIPs) which are aquaporin channels. Arsenic accumulation in plant roots results in the deceleration of root growth. Once these toxic ions translocate to the shoot, they begin to inhibit cellular metabolism which ultimately leads to plant death (Finnegan and Chen, 2012; Angulo-Bejarano et al., 2021).

Rice is the primary staple crop for more than 3.5 billion people worldwide, with 90% of it grown in Asia (U.S. Department of Agriculture). With up to 60 million people at risk of arsenic-related health conditions in Southeast Asia alone, finding effective strategies for mitigating the risk of consuming arsenic-rich rice is key to protecting global human health. Arsenic is considered a non-threshold human carcinogen by the International Agency for Research on Cancer (IARC), meaning that even small doses increase the risk of cancer (Signes-Pastor et al., 2016). Arsenic is associated with various cancers, including bladder, kidney, liver, lung, prostate, and skin cancers. Furthermore, arsenic exerts serious effects on many human biological systems, such as the gastrointestinal, hepatic, renal, cardiovascular, dermal, respiratory, and neurological systems (Prevention CfDCa).

Considering the importance of sustaining rice production and mitigating the risk of consuming arsenic-contaminated rice, several methods have been developed to quantify arsenic levels in crops (Ladha et al., 2021). Inductively coupled plasma-mass spectroscopy (ICP-MS), atomic absorption spectroscopy (AAS), and ion chromatography (IC) are commonly used to detect arsenic in plants (Wang et al., 2022). Although ICP-MS is a robust and reliable method to detect trace levels of elements, it suffers from several limitations (Spanu et al., 2019; Wilschefski and Baxter, 2019). Specifically, many different elements and compounds can cause signal interference with each other. Moreover, the complexity of ICP-MS analysis is costly and labor-intensive, requiring experienced laboratories to process samples. AAS has lower cost association per analysis and is also reliable, but equipment is equally expensive (Schneider et al., 2018). IC is generally the most cost-effective method but is inconsistent due to variations among column manufacturers while still requiring the involvement of an experienced laboratory. All these methods provide limitations to the average farmer who may not have the resources available to get crop samples analyzed by far-off laboratories. Lastly, the complicated pretreatment for these methods is destructive to the crop sample.

Numerous pieces of evidence show that Raman spectroscopy (RS) can be used for confirmatory diagnosis of biotic and abiotic stresses in plants (Altangerel et al., 2017; Egging et al., 2018). RS is an optical technique based on inelastic scattering caused by light interacting with molecules present in the sample (Orlando et al., 2021). The resulting spectra provide information about the molecular structure and composition of analyzed specimens. RS has shown promise for analyzing plant tissues, capable of diagnosing plant diseases, detecting environmental stress, and label-free phenotyping (Payne and Kurouski, 2021). This technique is primarily based on the relative concentrations of metabolites present within a plant, namely Raman active molecules phytochemicals such as terpenes, phenolics, and alkaloids. For example, a study in 2022 showed that RS could detect *Fusarium* head blight in wheat kernels due to the spectral changes in bands associated with lignin, carotenoids, pectin, cellulose, protein, and starch (Qiu et al., 2022). It was also recently demonstrated that RS could be used to diagnose nutritional deficiencies, salinity stress, and aluminum and iron toxicities with high accuracy in rice crops (Sanchez et al., 2020; Higgins et al., 2022a). Based on these previous findings, we hypothesize that RS can be used to detect changes caused by arsenic stress in rice.

In this study, we investigated the extent to which RS could be reliably used for the detection of arsenate and arsenite stresses within different cultivars of rice (*Oryza sativa*). We acquired Raman spectra from rice crops after their exposure to either arsenate or arsenite. Using partial least squares discriminant analysis (PLS-DA), we demonstrated that such spectra could be used for the confirmatory diagnosis of both arsenate and arsenite stresses in rice. We also performed ICP-MS to quantify the amount of arsenic accumulated by the rice crops. Finally, we utilized high-performance liquid chromatography (HPLC) analysis to reveal the plant metabolites sensed by RS. We found that RS detected changes in the concentration of lutein, ferulic acid, and vanillic acid due to the bioaccumulation of arsenic in rice.

## Materials and methods

### Experimental design

The rice plants were cultivated in a hydroponic system using plastic bins and Styrofoam boards. The Styrofoam boards were designed with circular holes for each rice plant, and a plastic mesh was securely attached to the bottom of each board panel. The rice seeds were pre-germinated before being placed into the hydroponics, with one seed per hole. For the performed experiments, 18 plants of each of the following cultivars were grown: IR64-Sub1A, IR154, Ciherang-Sub1A, and Presidio, **Supplementary Table S1**. These different cultivars were selected primarily to study the plant response to arsenic in a diverse array of rice. Presidio is a Southern U.S. *tropical japonica* rice cultivar while the rest were *indica* cultivars. The plants received nutrients via a Yoshida solution mixture consisting of macronutrients (114.30 mg/L NH_4_NO_3_, 50.40 mg/L NaH_2_PO_4_·2H_2_O, 89.30 mg/L K_2_SO_4_, 108.25 mg/L CaCl_2_ and 405 mg/L MgSO_4_·7H_2_O), and micronutrients [1.875 mg/L MnCl2·4H2O, 0.093 mg/L (NH_4_)_6_Mo_7_O_24_·4H_2_O, 1.09 mg/L H_3_BO_3_, 0.038 mg/L CuSO_4_·5H_2_O, 9.62 mg/L FeCl_3_·6H_2_O, 14.88 mg/L C_6_H_8_O_7_·H_2_O and 0.043 mg/L ZnSO_4_·7H_2_O] (Higgins et al., 2022a). The water and solution for the crops were completely replaced every 3 days, and the hydroponics were maintained at a pH of 5. Experimental growth conditions were controlled in a growth chamber set to a day/night cycle of 12h/12h, humidity to 55%, and day/night temperatures to 29°C/26°C (Higgins et al., 2022a). Rice was grown in such conditions for two weeks before initiation of arsenic stress.

The plants in the experiment were divided into three experimental groupings based on the oxidation state of arsenic administered: arsenite (As^3+^), arsenate (As^5+^), and the control grouping. This totaled 12 different groups of rice plants, accounting for the combinations of experimental conditions and cultivars. 50µM NaAsO_2_ was used for modelling arsenite stress, and 50µM Na_2_AsO_4_·7H_2_O was used for arsenate stress. The arsenic was added to the Yoshida solution for administration. Administration of the arsenic marked stress day 1.

### Raman spectroscopy and photography

For data collection, an Agilent Resolve hand-held Raman spectrophotometer was used to collect spectra from the rice leaves at 830 nm. Acquisition time was 1 second at a laser power of 495 milliwatts. 40 Raman spectra were acquired for each group of plants every other day, stopping at day 9. All spectra were baselined and normalized at the 1440 cm^-1^ peak.

Photographs of the crops were also collected at these time points. There were some visual differences between the arsenic-stressed crops and the control by day 5, noticeably in the number of leaves for each rice plant. Still, in the larger scope of a field containing arsenic, these differences were not strong enough to indicate high levels of arsenic stress (**Supplementary Figure 1**).

### Chemometrics

PLS_toolbox (Eigenvector Research Inc) was used in MATLAB to perform all statistical analyses. Data was downloaded from the instrument as CSV files then imported into MATLAB. First, ANOVA was performed for all peaks with visual change. Next, PLS-DA models were built for a binary comparison of each experimental group, with 2 to 6 latent variables used for each model. All HPLC and ICP-MS data were then checked for normality using QQ-plots, before performing unpaired two-tailed T-tests to measure significance.

### High-performance liquid chromatography

HPLC was performed to quantify the amount of carotenoids and phenylpropanoids in all groups of plants. Carotenoids were extracted by homogenizing 150 mg of rice leaf tissue with a mortar and pestle (Higgins et al., 2022b). 1.5 mL of a 1:2 v/v dichloromethane:chloroform mixture was added to the homogenized plant tissue, and the resultant solution was mixed on a thermomixer for 30 minutes at 4°C and 500 rpm. This agitation allowed for thoroughly mixing and extracting of the carotenoids from the plant tissue. The solution was then phase separated by adding 1 mL of 1 M NaCl before centrifugation for 10 minutes at 5,000 g. The aqueous and organic phases were separated, and 0.75 mL of the dichloromethane:chloroform mixture was added again to the aqueous phase. This portion then underwent a second centrifugation for 10 minutes at 5,000 g. The second organic phase from this portion was added to the original organic phase, and all aqueous phases were discarded. The organic phase was then dried in a Multivapor™ vacuum evaporator. Finally, the dried pellet was resuspended in 1 mL of methanol prior to HPLC injection. For phenylpropanoids, extraction began similarly by homogenizing 100 mg of rice leaf tissue with a mortar and pestle. 1.5 mL of methanol was then added to the homogenized plant tissue. The solution was sonicated for 1 hour to aid in efficient extraction. Both the phenylpropanoid and carotenoid samples were filtered with a 0.45 µm filter before injection.

The extracts were analyzed by reverse-phase HPLC using a C_30_, 3 μm column (250 × 4.6 mm) (Thermo Fisher Scientific Inc, part number 075723). We used a Waters 1525 pump equipped with the Waters 2707 autosampler and the 2489 Waters photodiode array detector. For carotenoid analysis, the mobile phases were (A) methanol:water (95:5, v/v) and (B) MTBE. The method for elution gradient began with 97% A until minute 6, where B increased from 3% to 100% linearly until minute 20. The flow then gradually returned to starting conditions at minute 23. The elution peaks were recorded at 450 nm. For phenylpropanoid analysis, the mobile phases were (A) 0.1% H_3_PO_4_ and (B) acetonitrile. The elution gradient method began at 95% A until minute 1. The gradient then decreased linearly to 40% A and 60% B until minute 13. The gradient rapidly dropped to 5% A over the next minute, before returning to starting conditions over the final minute. The elution peaks were recorded at 280 nm. All peaks were quantified for comparison by measuring the peak area under the curve (AUC) using Breeze software.

### Inductively coupled plasma mass spectroscopy

ICP-MS was performed to quantify the amount of arsenic present in each group. To prepare the sample, 200 mg of rice stem tissue was dried and then predigested in 10 mL of HNO_3_ in 50mL centrifuge tubes overnight. The next day, the tubes were placed in a water bath at 100°C for 3 hours to complete digestion. Beforehand, a hole was made in the cap of each tube to allow for ventilation, and the water bath was done under a running fume hood. The solutions were allowed to cool before 5 mL aliquots were taken from each tube. These aliquots were then diluted to 50mL for a 10x dilution for ICP-MS injection.

ICP-MS was run using a Quadrupole Inductively Coupled Plasma-Mass spectrometer (PerkinElmer NexION 300D) equipped with a Cetac ASX-520 autosampler. Argon was used as the carrier gas. Rhodium was used as an internal standard. The calibration curve for ICP-MS was generated using 1 g/L of certified reference material arsenic in 2% nitric acid. Dilutions of this external standard were made for 1 ng/mL, 25 ng/mL, 50 ng/mL, 100 ng/mL, and 200 ng/mL. All external standards and rice sample dilutions were made with ultrapure water.

## Results

### Raman spectroscopy

**Figure 1:**
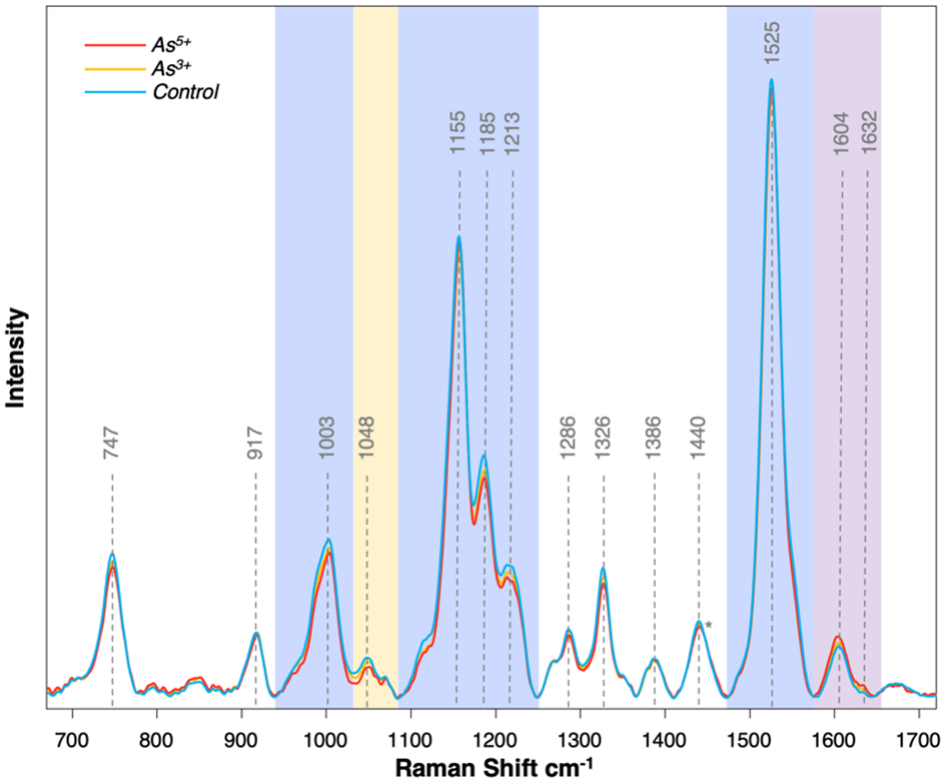
Average Raman spectra collected from each treatment group at experimental day 5. Blue regions correspond with carotenoids, purple region corresponds with phenylpropanoids, and yellow region corresponds with cellulose. Spectrum normalized at 1440 peak indicated by the asterisk (*).

The Raman spectra collected from rice leaves contained several peaks that corresponded to different biomolecules, such as carotenoids (1003, 1155, 1185, 1213, 1525 cm^-1^), phenylpropanoids (1604, 1632 cm^-1^), cellulose (917, 1048 cm^-1^), carbohydrates (1155 cm^-1^), and pectin (747 cm^-1^) (Sanchez et al., 2020; Higgins et al., 2022a, Higgins et al., 2022b) (**Figure 1**). The peaks at 1286, 1326, 1386, and 1440 cm^-1^ could be assigned to CH and CH_2_ vibrations, which are present in nearly all classes of biological molecules. Therefore, these vibrations cannot be assigned to a specific class of molecular analytes.

**Figure 2:**
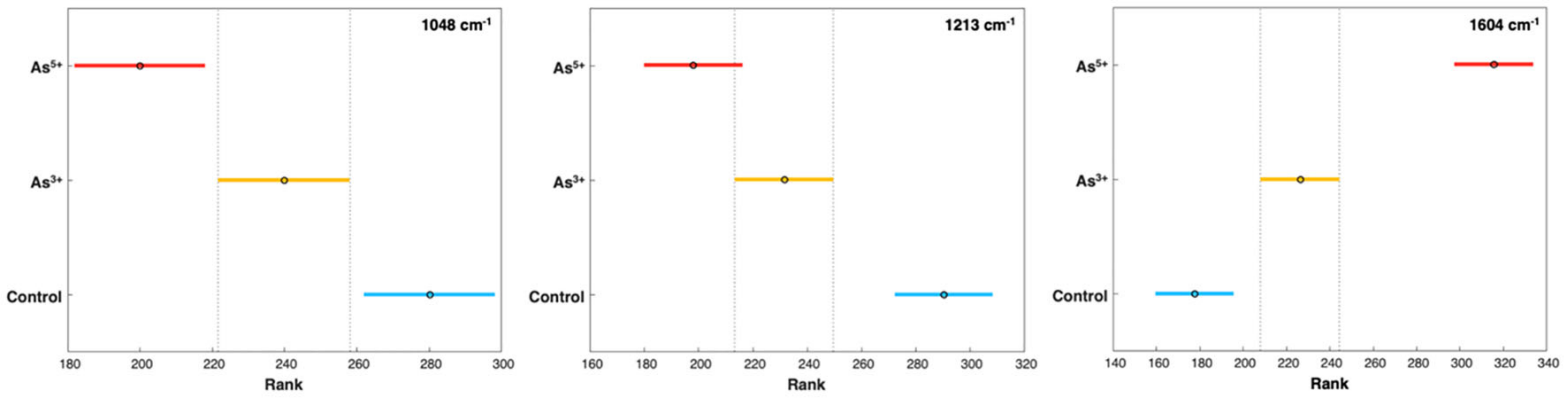
Post-hoc comparison using the Tukey HSD after one-way ANOVA. Graph indicates differences in average peak intensity on experimental day 5 at 1048 cm^-1^, 1213 cm^-1^, and 1604 cm^-1^, corresponding to cellulose, carotenoid, and phenylpropanoid content respectively. As3+ and As5+ are statistically significant from control and from each other, except at the 1213 cm-1 peak.

In the Raman spectra acquired from rice plants exposed to arsenic, we observed substantial changes in the intensities of the discussed above vibrational bands. We primarily observed a decrease in intensity of carotenoid vibrations and an increase in intensity of phenylpropanoid vibrations. A slight decrease was also noticed in vibrational bands that could be assigned to cellulose. These findings indicate that arsenic toxicity in rice is linked to a reduction in the concentration of carotenoids (**Figure 2**). These molecules are crucial in photosynthesis and photoprotection by protecting the plant against reactive oxygen species (ROS) generated by chlorophyll under high light conditions (Khorobrykh et al., 2020; Swapnil et al., 2021). This protective mechanism involves carotenoid degradation, such as the enzymatic oxidation of neoxanthin to abscisic acid. This molecular analyte triggers the plant’s immune response to both biotic and abiotic stresses (Havaux, 2014; Stanley and Yuan, 2019). The predominant plant carotenoid is lutein, which serves a crucial function in the assembly and preservation of photosystems (Lv et al., 2012). Decreased concentrations of lutein are associated with oxidative damage due to light stress inducing more ROS than a plant can process. Given that arsenic also induces intracellular ROS production, it is reasonable to anticipate that rice would respond in a similar way as it does to light stress by utilizing its carotenoids (Hu et al., 2020).

Our findings also showed that arsenic toxicity caused an increase in the concentration of phenylpropanoids in rice (**Figure 2**). Phenylpropanoids are a large class of biomolecules found in plants. These molecules are the major constituents of lignin, and they protect plants against UV light and pathogens (Deng and Lu, 2017). In the Raman spectra acquired from rice plants exposed to arsenic, we observed an increase in the intensity of the 1604 cm^-1^ peak compared to the intensity of this band in the spectra acquired from the leaves of healthy plants. These spectroscopic changes point to the production of phenylpropanoids by rice as a response to arsenic stress.

Phenylpropanoids, especially phenolic acids and flavonoids, are known to increase in content during abiotic stress events due to activation of phenylpropanoid biosynthetic pathways (Sharma et al., 2019). Elevated levels of phenolic compounds can adversely affect plant growth, as observed in our experimental crops on day 9 (Cheynier et al., 2013). Functionally, these phenolic compounds possess antioxidant properties and play a vital role in regulating immune responses. Arsenic, as an exogenous source of ROS, can easily induce the biosynthetic enzymes responsible for phenylpropanoid production. In addition, researchers have identified that As can cause biochemical changes in the composition of cellulose and lignin in elm, both critical components to cell wall’s structure (Waliszewska et al., 2019).

Furthermore, a slight decrease in the intensity of the 1048 cm^-1^ peak was noticed in the spectra acquired from the rice exposed to arsenic [**Figure 2**]. These spectroscopic changes point to a decrease in cellulose content upon arsenic-induced toxicity. Previous research has also shown that high levels of arsenic could cause a reduction in the cellulose content of the cell wall (Huang et al., 2021). Based on these results, we conclude that arsenic induces structural changes to the cell walls of rice plants.

### High-performance liquid chromatography

**Figure 3:**
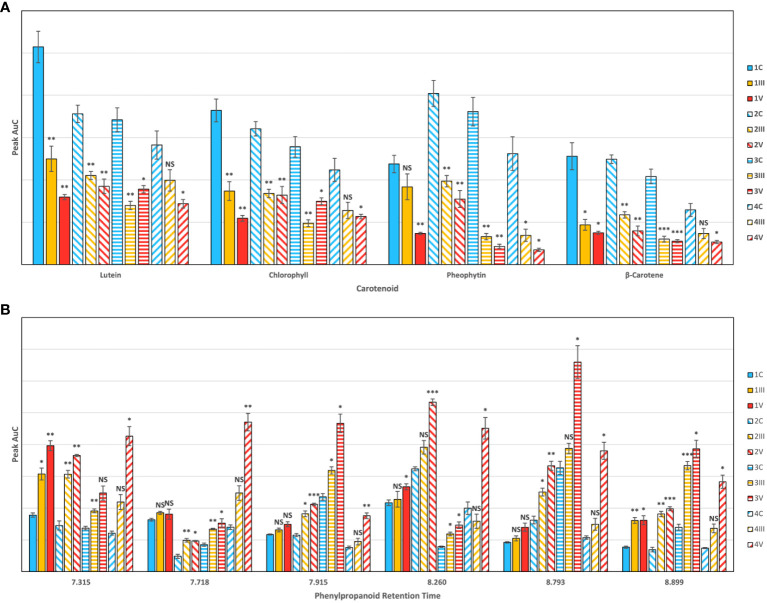
HPLC results for **(A)** carotenoids and **(B)** phenylpropanoids, by cultivar and experimental condition, indicating average peak area with standard error bars. Cultivars are numbered as (1) IR64-Sub1A (2) IR154 (3) Ciherang Sub-1A (4) Presidio. Conditions are C control (III) As3+ (V) As5+. Labels indicate T-test results for the comparison of each arsenic group versus the control: NS is no significance, * is P ≤ 0.05, ** is P ≤ 0.01, and *** is P ≤ 0.001.

As was discussed above, spectroscopic analysis of the rice leaves exposed to both arsenate and arsenite stresses indicated a decrease in the concentration of carotenoids, with a simultaneous increase in the concentration of phenylpropanoids. We used HPLC to validate these expectations as to identify the specific molecular analytes that were sensed by RS in the non-invasive detection and identification of arsenic stress in rice (**Figure 3**).

HPLC analyses of plant leaf extracts revealed four prominent peaks that could be assigned to lutein (RT = 12.14), chlorophyll (RT = 14.85), pheophytin (RT = 16.52), and β-carotene (RT = 19.86) respectively (Dou et al., 2021; Higgins et al., 2022b). We observed a significant decrease in intensity of all peaks in the samples extracted from the leaves of rice plants exposed to arsenic. Furthermore, we found that arsenate-induced toxicity caused more substantial changes in the carotenoid concentration than arsenite-induced stress. We also observed some variation in the intensity of peaks that corresponded to various carotenoids in the chromatograms of different cultivars. Based on these changes, we could conclude that Presidio suffered more from arsenic stress than the three Asian cultivars. Among the four measured carotenoids, previous work has shown that lutein is the primary carotenoid detected at 830 nm (Dou et al., 2021), since chlorophyll and pheophytin are highly fluorescent (**Supplementary Figure 2**). Coupled with our HPLC data, we can conclude that changes directly related to lutein content are responsible for the carotenoid intensity decreases we detect in rice crops.

The chromatogram for phenylpropanoids contained over 20 peaks, so we narrowed down our analysis to the six most prominent peaks. HPLC previously done in rice has shown an increased synthesis of phenolic compounds as a response to stress in certain rice strains (He et al., 2012). We found that arsenic stress statistically increased phenylpropanoid content in at least one peak for every cultivar and visually increased at most peaks. Several peaks in the As^3+^ group did not show a statistically distinct response from the control. There was also much more variability in phenylpropanoid content between cultivars as compared to looking at carotenoids (**Supplementary Figure 3**). This indicates that certain cultivars 1) have different base levels of phenylpropanoid production and 2) have differing responses in levels of phenylpropanoid synthesis as a response to stress. This can be illustrated by looking at the peak with RT = 7.915 min, where IR64-Sub1A had little increased production because of arsenic stress; however, Ciherang-Sub1A had a greatly augmented production of phenylpropanoids. There are variations in arsenic resistance among different rice cultivars, and these variations in phenylpropanoid expression may contribute to the differing levels of resistance observed in each cultivar.

### Inductively coupled plasma mass spectroscopy

**Figure 4:**
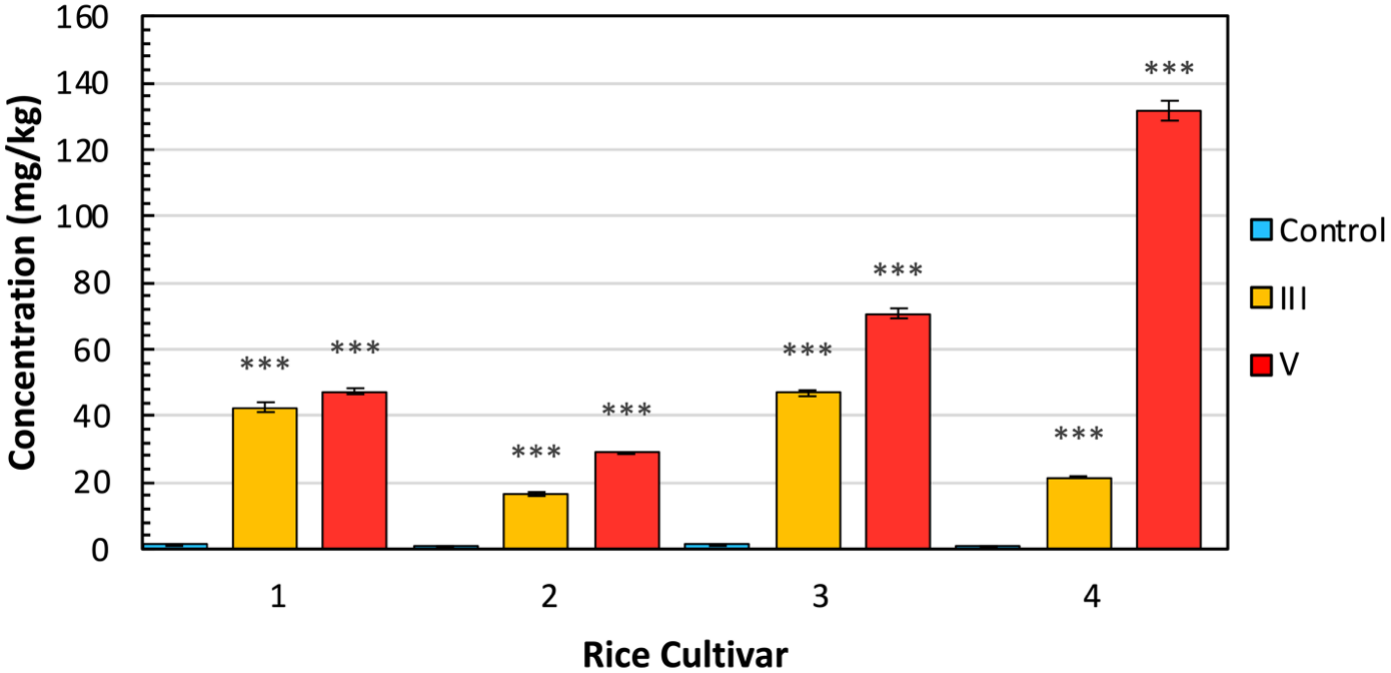
ICP-MS results by cultivar and experimental condition, indicating average concentration with standard error bars. Cultivars are (1) IR64-Sub1A (2) IR154 (3) Ciherang Sub-1A (4) Presidio. Conditions are (C) control (III) As3+ (V) As5+. Labels indicate T-test results for the comparison of each arsenic group versus the control: NS is no significance, * is P ≤ 0.05, ** is P ≤ 0.01, and *** is P ≤ 0.001.

Traditionally, ICP-MS is used to quantify the amount of arsenic found within rice crops. Here, we performed ICP-MS to validate the stress detected by RS with actual arsenic accumulation (**Figure 4**). This also allowed us to investigate differences in arsenic uptake by different cultivars. We found that an exceptional amount of arsenic accumulated in the arsenic-stressed rice compared to the control. Presidio also accumulated a much greater amount of arsenic under stress in comparison to the three Asian cultivars. Still, these values align with other studies on arsenic accumulation in rice. Despite the elevated levels of arsenic found within plant tissues, rice grains typically exhibit a prevalence of lower concentrations of inorganic arsenic in contrast to organic arsenic. Furthermore, the total arsenic content within rice grains remains notably reduced in comparison to other plant tissues.

## Discussion

**Figure 5:**
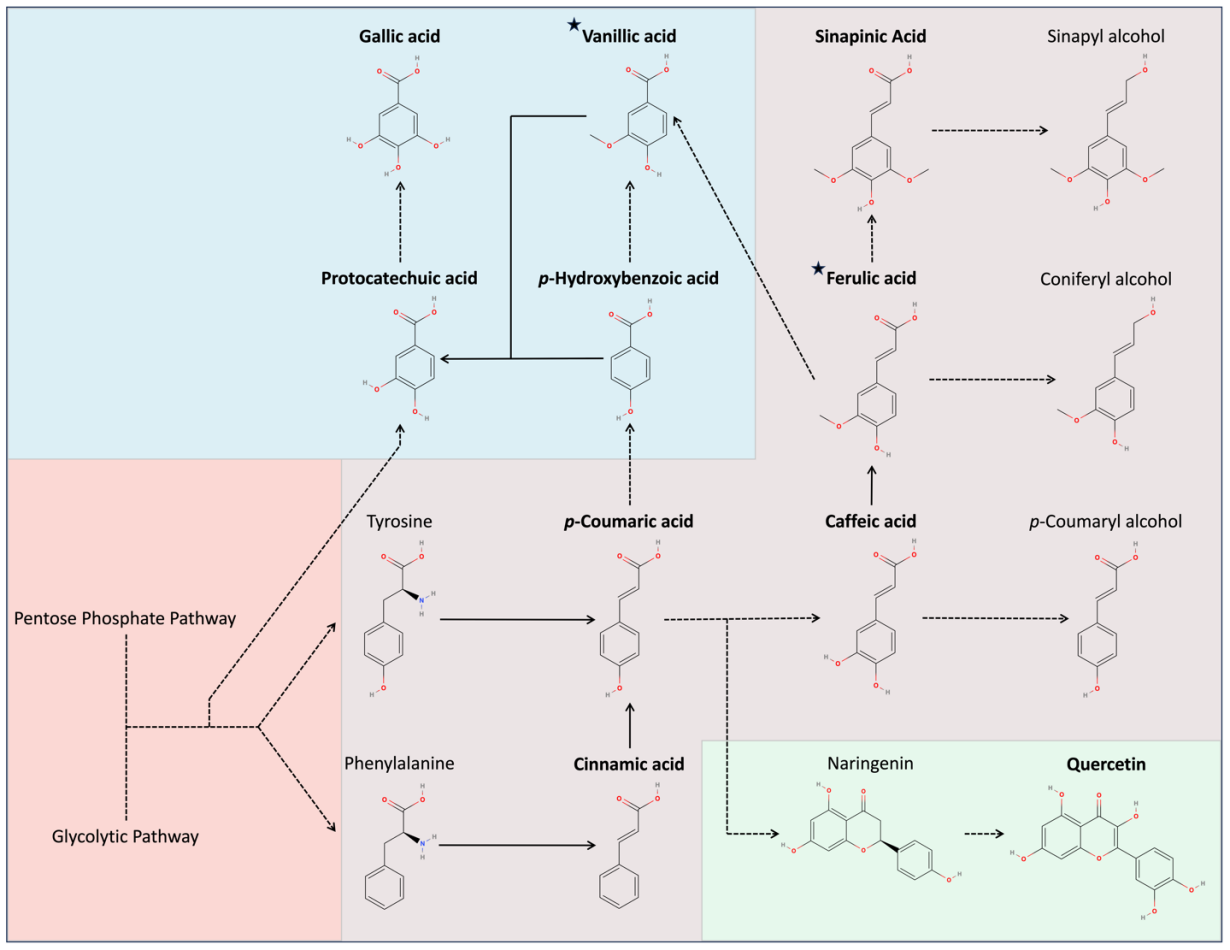
Secondary metabolite pathways in *Oryza sativa*. Red indicates non-secondary metabolites, purple indicates hydroxycinnamics, blue indicates hydroxybenzoics, and green indicates flavonoids. Bolded text indicates compounds tested, while stars indicate compounds identified.

In comparison to carotenoids, the plants exhibit an immense diversity and number of phenylpropanoids. These molecules can be categorized according to their structure and involvement in different metabolic pathways. One of the more important pathways is the phenylpropanoid pathway (Biała and Jasiński, 2018). Lignin biosynthesis occurs through this pathway via the polymerization of three monomeric units: p-coumaryl alcohol, coniferyl alcohol, and sinapyl alcohol (Fraser and Chapple, 2011). Since lignin increases during oxidative stress, we investigated this pathway to identify if it was responsible for the changes within the Raman spectra. We accomplished this by conducting HPLC on five hydroxycinnamate standards found within the pathway (cinnamic acid, *p*-coumaric acid, caffeic acid, ferulic acid, and sinapinic acid) (**Figure 5**). Hydroxycinnamates are central intermediates in phenylpropanoid pathway that are directly converted into lignin (Ralph, 2010). To rule out other pathways, we also tested four hydroxybenzoates (gallic acid, vanillic acid, *p*-hydroxybenzoic acid, and protocatechuic acid), and two flavonoids (quercetin and rutin) to rule out other pathways. Hydroxybenzoates are synthesized through shikimate and phenylpropanoid pathways, serving as antioxidants and structural components (Deng and Lu, 2017). In contrast, flavonoids branch into distinct pathways within the overall phenylpropanoid pathway, acting as pigments and signaling molecules (Deng and Lu, 2017).

Among the six prominent phenylpropanoid peaks we reported, the retention times of vanillic acid and ferulic acid corresponded with two specific peaks, RT = 7.315 min and RT = 8.899 min, respectively. Metabolically, ferulic acid is a precursor to the monolignol coniferyl alcohol, possibly indicating increased lignin production. Vanillic acid is not a hydroxycinnamate but is a hydroxybenzoate derived from several intermediates in the phenylpropanoid pathway. It plays a diverse protective role with strong antioxidant properties and an ability to upregulate components of the antioxidant system (Parvin et al., 2020). None of the other injected standards corresponded with any major peaks observed.

**Figure 6:**
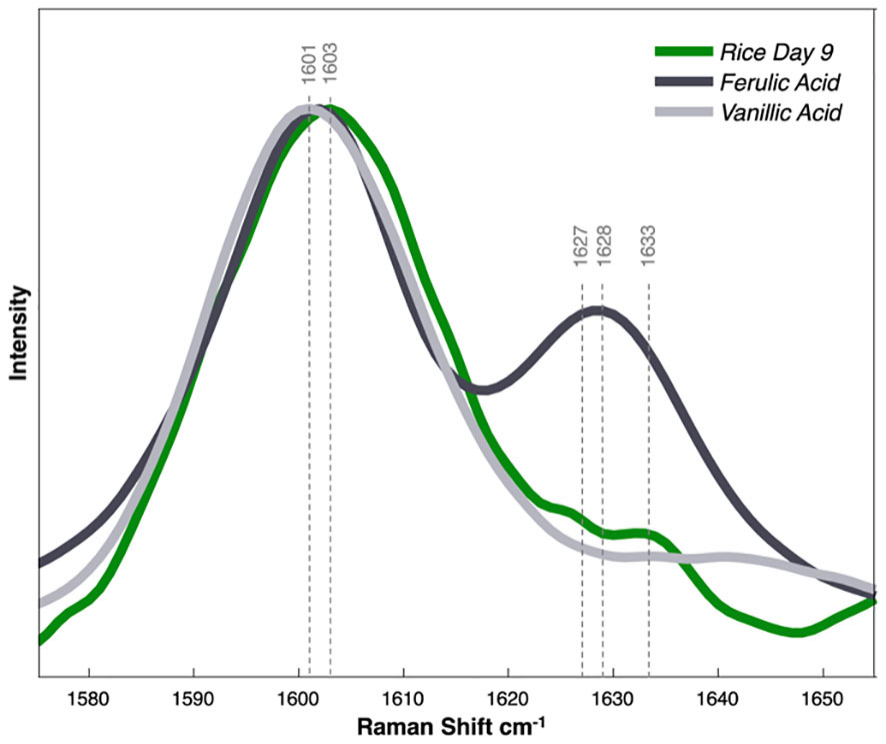
Raman spectra collected from phenylpropanoid standards of ferulic/vanillic acid dissolved in water. Normalized by peak height.

To compare our HPLC findings with the observed spectra, we acquired Raman spectra from powdered forms of vanillic acid and ferulic acid (**Figure 6**). Notably, the 1601 cm^-1^ peak observed in the spectra of the two standards closely matched the 1603 cm^-1^ peak in the spectra of the rice on day 9. In the spectra of ferulic acid, a secondary peak was observed at 1628 cm^-1^, aligning with a minor shoulder evident in the rice spectra. It is important to note that the Raman spectra of the arsenic-stressed groups exhibited non-uniformity within the phenylpropanoid region, characterized by several shoulders. This differs from the symmetric peaks observed in the control spectra. Moreover, over the 9 days of stress, the primary phenylpropanoid peak shifted from 1607 cm^-1^ to 1603 cm^-1^. This implies the involvement of multiple phenylpropanoid species contributing to the overall spectral profile, and that during arsenic stress, the predominant phenylpropanoid species is likely changing. Nevertheless, the observed increases in the chromatographic peaks and the corresponding Raman spectra of the standards lead us to conclude that vanillic acid and ferulic acid significantly contribute to the changes noticeable within the Raman spectra acquired from plant leaves.

To verify the statistical significance of changes within the Raman spectra both ANOVA (analysis of variance) and PLS-DA were performed. The 1048 cm^-1^, 1213 cm^-1^, and 1604 cm^-1^ peaks best demonstrated the changes in biomolecular content. At the cellulose and carotenoid peaks, the differences in intensity from the control were statistically significant by experimental day 5; however, in the phenylpropanoid peak at 1604 cm^-1^, the increases in peak intensity were significant as early as day 3 (**Figure 2**). It was noted in the control group that both carotenoid and phenylpropanoid content gradually increased over the experimental days; however, given the young age of the rice crops, this was most likely simply due to plant development.

We used PLS-DA to quantify the accuracy of RS as a diagnostic method for detecting arsenic stresses. PLS-DA is a multivariate dimensionality-reduction tool that uses a supervised machine learning algorithm to classify large sets of data (Ruiz-Perez et al., 2020). The model was trained using 40 spectra acquired from each group of plants. Next, we tested the model’s ability to differentiate the spectra collected from plants exposed to arsenite and arsenate and from the control plants. We evaluated the ability of RS to differentiate arsenic stress from the control (sensitivity) and arsenite stress from arsenate stress (selectivity). Our findings showed high reliability of RS already by day 1 of stress treatment (**Table 1**). At this time point, RS coupled to PLS-DA could detect arsenate and arsenite stresses with 96% and 76%, respectively. Averaged across the five time points, the model could detect the arsenic stresses with 89% accuracy. PLS-DA results also showed that RS could be used to differentiate between arsenate and arsenite stresses with 82% accuracy, although this value decreased as the experiment progressed.

**Table 1:** PLS-DA true predication rates (TPR) for each treatment and experimental time point.

Day	1	3	5	7	9
As^5+^ Sensitivity	92%	87%	96%	93%	98%
As^3+^ Sensitivity	76%	74%	88%	93%	93%
Selectivity	82%	84%	84%	71%	72%

The PLS-DA model uses latent variables for its predictive power, and plotting these orthogonal factors allow us to visualize how the model classifies these spectra based on their similarities and differences. PLS-DA results showed that the sensitivity of RS in differentiating arsenic stress from the control increased from day 1 to day 9. However, we found that the selectivity of RS in differentiation between arsenite and arsenate stresses progressively decreased as the time of plant exposure to both stressors increased. By day 9, the LVA plot showed substantial overlap between the two arsenic stress groups, whereas in the control group, there was only partial overlap (**Supplementary Figure 4**). This decrease in the selectivity is expected since high levels of arsenic stress will ultimately lead to crop mortality, regardless of the oxidation state of arsenic. By observation, Ciherang-Sub1A and Presidio cultivars looked unhealthy and close to dying on day 9 for both arsenic stress groups, while the two other cultivars survived much better under arsenic stress.

**Figure 7:**
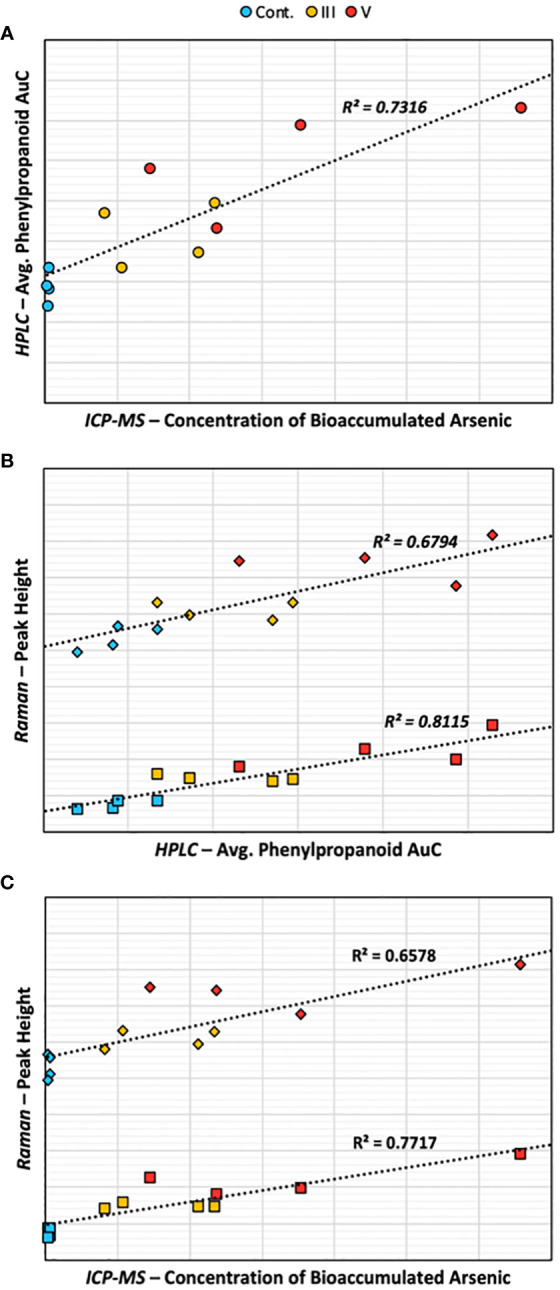
Scatter plots comparing **(A)** HPLC and ICP-MS, **(B)** Raman and HPLC, and **(C)** Raman and ICP-MS. Diamonds represent the 1604 Raman peak, squares represent the 1632 Raman peak, circles represent the average AuC of the reported phenylpropanoid peaks.

Lastly, recognizing the impracticality of employing all three analytical methods for routine sample testing, we examined the interrelations among the results of each method utilized. Scatter plots were generated for each combination of RS, HPLC, and ICP-MS, taking into account all relevant peaks from the chromatograms and Raman spectra (**Supplementary Figure 5**). Notably, an increase in average phenylpropanoid concentration, as identified by HPLC, closely aligned with the arsenic bioaccumulation detected by ICP-MS (**Figure 7**). The rise in average phenylpropanoid concentration also strongly correlated with the increase in phenylpropanoid peak intensity detected by RS, confirming our observations highlighted in the HPLC section. In the last comparisons, we observed that the intensities of the two phenylpropanoid Raman peaks correlated with arsenic bioaccumulation. Notably, the 1632 cm^-1^ peak demonstrated a stronger correlation than the 1604 cm^-1^ peak, suggesting that the increase in this shoulder peak serves as a superior indicator for both phenylpropanoid concentration and arsenic bioaccumulation. In addition, the relationship between phenylpropanoid concentration measured by HPLC and ICP-MS was most pronounced in the peak at RT=7.718. In contrast, the strongest correlation between HPLC and Raman spectroscopy was identified in the peak at RT=7.315, corresponding to vanillic acid. These correlations provide crucial insights into how our RS findings align with conventional analytical techniques. However, it is important to acknowledge the limitations of these findings due to our dataset size. Future studies could greatly enhance these correlations by growing rice in varying concentrations of arsenic, facilitating the development of robust calibration curves. Furthermore, considering the complex field conditions impacting rice development, more research regarding RS’s sensitivity in detecting arsenic under these conditions must be done.

## Conclusion

RS serves as a valuable tool for the non-invasive and non-destructive identification of arsenic stress in rice crops. The spectral changes observed, including decreased carotenoid intensity and increased phenylpropanoid concentration, provide clear markers of arsenic-induced physiological alterations in the plants. The successful detection of arsenate and arsenite stresses when coupled with PLS-DA in the early stages of exposure was also validated with HPLC. We found that vanillic acid and ferulic acid contribute substantially to the spectral changes observed in the phenylpropanoid region in the Raman spectra. Overall, this research offers several insights into RS as a strategy for mitigating arsenic-related health risks in crops. This transition towards digital farming will aid agronomists in the early detection of arsenic, allowing them to prevent contaminated crops from reaching the market early on. The brief two-week timeframe of this experiment even indicates its potential promise for early screening of resistant rice germplasm. Looking forward, future studies should focus on determining RS’s limit of detection for arsenic stress in complex environments to further refine its potential for usage in agriculture settings.

## Data availability statement

The raw data supporting the conclusions of this article will be made available by the authors, without undue reservation.

## Author contributions

IJ: Conceptualization, Data curation, Formal analysis, Investigation, Methodology, Validation, Visualization, Writing – original draft, Writing – review & editing. TD: Investigation, Methodology, Writing – review & editing. SB: Investigation, Methodology, Validation, Visualization, Writing – review & editing. ES: Project administration, Supervision, Writing – review & editing. DK: Conceptualization, Funding acquisition, Project administration, Resources, Supervision, Writing – original draft, Writing – review & editing.
