# Supplementary material for: Diagnosing arsenic-mediated biochemical responses in rice cultivars using Raman spectroscopy
Source: Front Plant Sci. 2024 Mar 25;15:1371748. doi: 10.3389/fpls.2024.1371748 (PMC10999542; doi:10.3389/fpls.2024.1371748)
Supplement: Supplementary file 1 [file DataSheet_1.docx]

**Diagnosing arsenic-mediated biochemical responses in rice cultivars using Raman spectroscopy**

*Isaac D. Juárez^1,2^, Tianyi Dou^1^, Sudip Biswas^3^, Endang Septiningsih^3^, Dmitry Kurouski^1,2^**

1. Department of Biochemistry and Biophysics, Texas A&M University, College Station, Texas 77843, United States
2. Interdisciplinary Faculty of Toxicology, Texas A&M University, College Station, Texas 77843, United States
3. Department of Soil and Crop Sciences, Texas A&M University, College Station, Texas 77843, United States

Corresponding author: Dmitry Kurouski; Email: dkurouski@tamu.edu

Supporting Information

**Table S1**: Cultivars used in our study and their region of origin.

| IR64-Sub1A | Asia |
| --- | --- |
| IR154 | Asia |
| Ciherang-Sub1A | Asia |
| Presidio | Texas, USA |


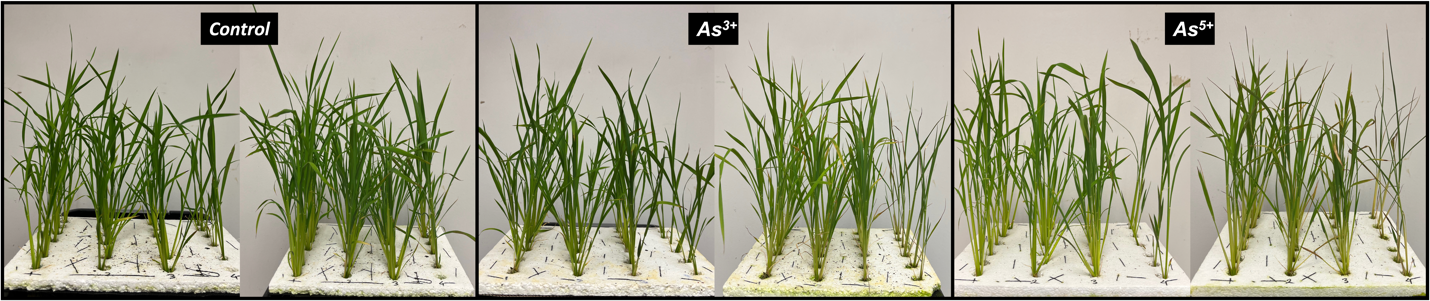


**Figure S1:** Rice crops at experimental day 1 and day 5 of Arsenic stress. Experimental day goes 1 to 5 left to right by photograph; Cultivars go from 1 to 4 left to right by column.


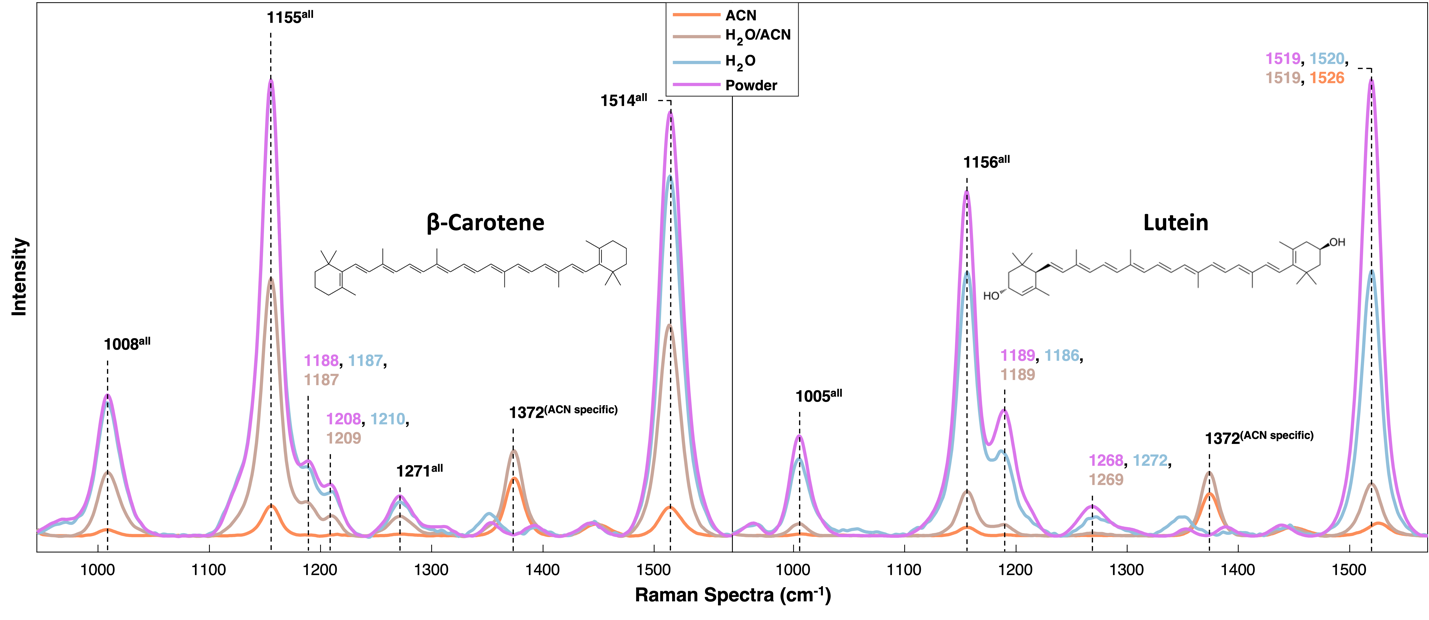


**Figure S2:** 0.45mM carotenoid standard solutions prepared in acetonitrile (ACN), deionized water (H_2_O), and a 1:1 mixture of ACN/H_2_O.

**Figure S3.** Results of high-performance liquid chromatograph for each experimental group. The first relevant peak is marked with red. Details regarding peak integration can be found in the materials section.

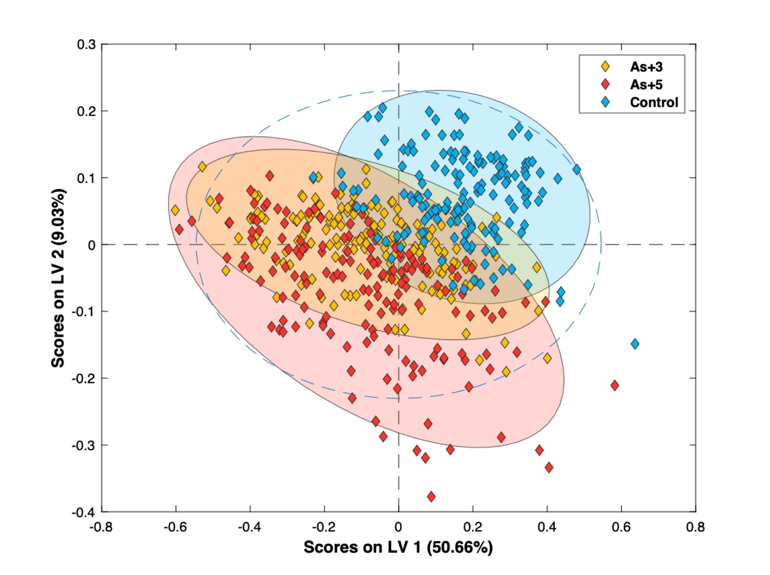


**Figure S4:** Latent variable analysis (LVA) plot for experimental day 9. These two variables represent around 60% of the separation, while the full binary model used an average of six variables.


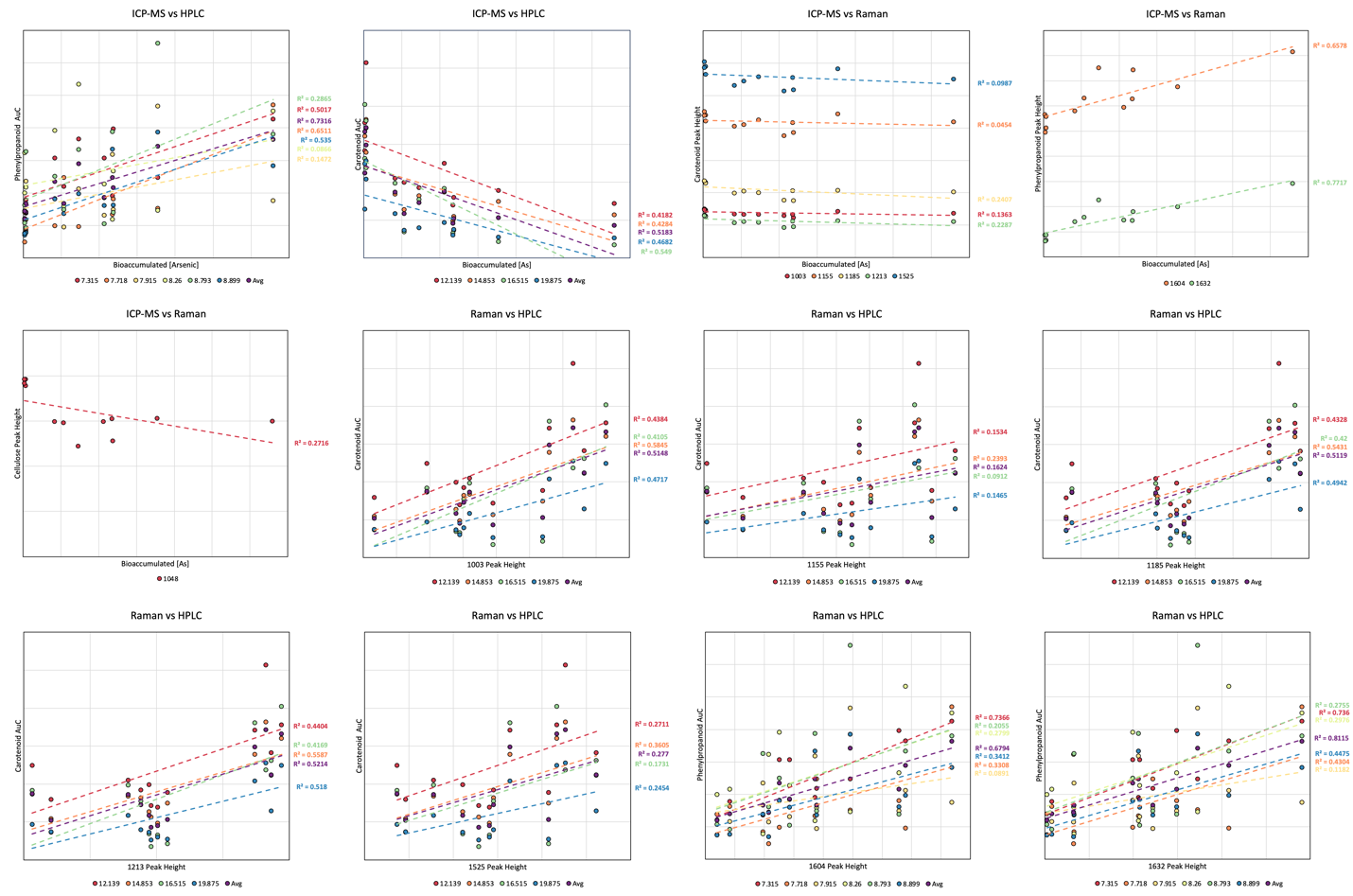


**Figure S5.** Scatter plots comparing each analytical method, accounting for different Raman peaks, HPLC retention times, and ICP-MS arsenic concentrations.
